# Intelligent Fault Diagnosis of HVCB with Feature Space Optimization-Based Random Forest

**DOI:** 10.3390/s18041221

**Published:** 2018-04-16

**Authors:** Suliang Ma, Mingxuan Chen, Jianwen Wu, Yuhao Wang, Bowen Jia, Yuan Jiang

**Affiliations:** School of Automation Science and Electrical Engineering, Beihang University, Beijing 100191, China; masuliang@buaa.edu.cn (S.M.); mingxuan_chen@buaa.edu.cn (M.C.); 18810371198@163.com (Y.W.); jiabowen109@126.com (B.J.); jiangy_luckystar@163.com (Y.J.)

**Keywords:** high-voltage circuit breakers, mechanical fault diagnosis, wavelet packet decomposition, random forest algorithm, ensemble learning, feature space optimization

## Abstract

Mechanical faults of high-voltage circuit breakers (HVCBs) always happen over long-term operation, so extracting the fault features and identifying the fault type have become a key issue for ensuring the security and reliability of power supply. Based on wavelet packet decomposition technology and random forest algorithm, an effective identification system was developed in this paper. First, compared with the incomplete description of Shannon entropy, the wavelet packet time-frequency energy rate (WTFER) was adopted as the input vector for the classifier model in the feature selection procedure. Then, a random forest classifier was used to diagnose the HVCB fault, assess the importance of the feature variable and optimize the feature space. Finally, the approach was verified based on actual HVCB vibration signals by considering six typical fault classes. The comparative experiment results show that the classification accuracy of the proposed method with the origin feature space reached 93.33% and reached up to 95.56% with optimized input feature vector of classifier. This indicates that feature optimization procedure is successful, and the proposed diagnosis algorithm has higher efficiency and robustness than traditional methods.

## 1. Introduction

As a piece of control and protection equipment, high-voltage circuit breakers (HVCB) play an important role in the power system. Various research has reported that the mechanical faults of HVCB will give rise to significant harm and outage costs [1]. Therefore, a handful of researchers have made efforts to analyze failure cause and optimize the structure of HVCB. With the development of machine learning development, intelligent diagnosis technology has been successfully applied in various fields. On this basis, establishing an intelligent identification model for HVCB mechanical fault has become an irresistible trend.

The contact displacement and electromagnet coil current of a closing or opening process have been used as typical information for distinguishing the operation condition of HVCB. For example, Ali Forootani et al. [2] established a dynamic model using Lagrange’s method, and analyzed the resulting faults in the travel curve. An image matching-based algorithm integrating diamond search strategy was proposed to avoid the deficiencies of conventional travel-time curve measurement. This algorithm has been successfully applied in detection of mechanical properties with extreme learning machines [3]. On-line hybrid fault diagnosis model based on electromagnet coil current information was also developed [4,5]. Recently, a considerable amount of literature has been reported on the vibration characteristics of HVCB [6,7,8]. All this research indicates that the vibration signal of HVCB during operation is non-stationary and non-linear, which is more difficult to capture.

Time-frequency analysis tools have been widely utilized in extracting the fault feature vector of the vibration signal. Developed by Huang et al. in 1998, empirical mode decomposition (EMD) has become one of the most potent time-frequency analysis technologies. Based on the local characteristic time scale of a signal, it can decompose complicated signals into a number of intrinsic mode functions (IMFs) containing the characteristic information of the original signal [9,10]. However, the conventional EMD method is subjected to disadvantages such as end effect, high computational complexity and inconformity of decomposition among different signals, spectrum overlapping, and chaotic boundary frequency among different intrinsic modes. Therefore, numerous experts have put forward a series of improved methods, such as local mean decomposition (LMD) [6], improved ensemble empirical mode decomposition (EEMD) [11], vibration mode decomposition (VMD) [12] and so on. These improved approaches can successfully address spectrum confusion problems but are subjected to some other weaknesses. In contrast to the EMD approach, wavelet transform (WT) was proposed as an adjustable windowed Fourier transform with limited energy leakage [13]. Moreover, WT was extended to wavelet packet transform (WPT) to effectively split the high-frequency band containing rich fault modulation information [14]. Once wavelet basis function and decomposition scale are determined, the results of wavelet package transform will be the signal under a certain scale, whose frequency components are only related to the sample frequency rather than the signal itself. Obviously, wavelet analysis is not a self-adaptive signal processing method in nature [9,12]. Owing to excellent adaptive and multi-resolution capability, WPT is still regarded as a powerful mathematical tool in handling vibration signal in various fields of engineering [15,16].

After extracting the feature vector of vibration signal using time-frequency analysis tools, building a high-accuracy and robust classifier becomes a key issue. Hussain et al. designed an expert system consisting of a signal processor module and a factual database module to diagnose the faults of power circuit breakers [17]. For the case of small sample, support vector machine (SVM) and its improved method have been applied for identifying mechanical faults of HVCBs, and satisfactory diagnostic performance can be achieved [18,19,20]. Along with artificial intelligence technology and meta-heuristic approaches widely applied to control field [21,22,23,24], a novel method based on particle swarm optimization-support vector domain description and kernel-based fuzzy c-means was proposed for adaptive fault diagnosis of HVCBs under real-time updating of model classification and lack of knowledge acquisition [25]. In addition, there are masses of categorizers practiced in various scenarios. For instance, decision tree classifiers can diagnose motor fault tasks or detect breast cancer based on medical data [26,27,28]; naive Bayes algorithm has been applied as a fault classifier to investigate the status of a monoblock centrifugal pump or an engine [29,30,31].

Generally speaking, the above literature discusses powerful detectors for diagnosis. Obviously, the training process of a single classifier is affected by global error rate, so the models may be biased towards the majority class and ignore the minority class. Additionally, some abnormal collected samples or the dispersion of the dataset will potentially cause over-fitting [32]. Ensemble learning, which is common in machine learning, can enhance the performance of identification systems by constructing a set of individuals and weak classifiers and combining them to classify or predict new data with weighted or unweighted voting method [33]. A novel intelligent method with multivariable ensemble-based SVM was verified by detecting complex faults of different severity levels in the locomotive roller bearings [34]. Proposed by Breiman [35] and studied by Biau et al. [36], the random forest is an improved machine learning algorithm based on random subspace and ensemble learning [37]. In real industrial environments, it is difficult to acquire a large number of samples for classifier training and estimate the significance of feature candidates for fault diagnosis. Under this circumstance, RF-based models have better performance than other classical classifiers [38,39,40].

In this paper, a new feature indicator integrating wavelet package transform (WPT) was proposed, and the diagnosis model was designed and optimized based on random forest for diagnosing HVCB mechanical faults. Compared with the traditional feature indicators such as wavelet time-frequency entropy as well as general classification methods such as decision tree (DT) [28], naive Bayes classifier (NB) [29], one-class support vector machine (OCSVM) [20] and bagging tree (BT) [33], the contributions of this paper can be summarized as follows.

(1) HVCB mechanical fault is diagnosed by a holistic approach which integrates vibration signal acquisition system, feature extraction based on WPT and optimal classifier with random forest;
(2) Through comparing the depicted level of vibration information to that of time-frequency entropy, this paper presents time-frequency wavelet energy rate (WTFER) based on WPT and realizes full-scale description of vibration signal;
(3) The importance of feature variable is evaluated using data out of bagging based on ensemble learning. An optimal classifier based on random forest algorithm is designed to diagnose HVCB faults.

The remainder of this paper is organized as follows: Section 2 presents the procedure of the identification system based on the vibration of HVCB and experiment platform; Section 3 discusses vibration signal time-frequency feature extraction using wavelet package transform. Section 4 discusses the establishment of HVCB fault classifier and the optimization of feature space. Section 5 compares the optimal RF identification model with other traditional diagnosis methods to highlight the advantageous accuracy for optimal RF identification model. Finally, Section 6 concludes the contributions of this paper.

## 2. Identification System and Experiment Platform

### 2.1. The Identification System

**Figure 1:**
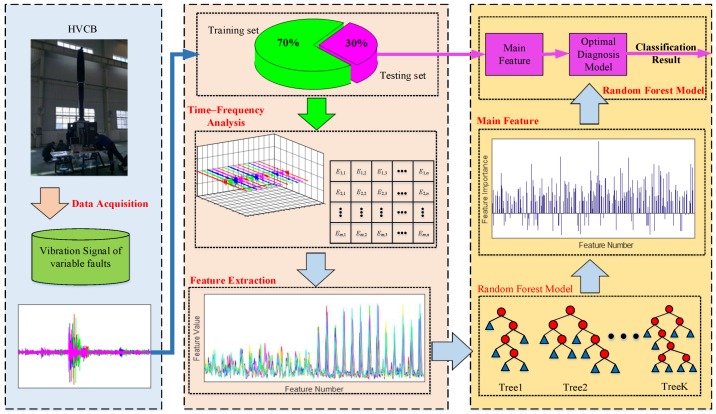
Structure flow of identification process.

In this study, the fault diagnosis process of HVCB based on vibration signal includes three parts: data acquisition under variable faults, feature extraction using wavelet package transform (WPT) for time-frequency analysis, and optimal identification model based on random forest (RF), as shown in Figure 1. Table 1 shows the general procedure of HVCB fault detection by the proposed method.

As seen from Figure 1, 70% of measured data was applied for designing the random forest diagnosis model, while the remaining 30% of data was used for measurement of fault diagnosis accuracy. In this study, we tested the vibration signals of HVCB under 6 conditions (i.e., Normal case, Fault I—closing spring fatigue, Fault II—opening spring fatigue, Fault III—damping increasing for transmission shaft, Fault IV—oil damper leaking, Fault V—looseness of base screw), with 50 samples per each condition, and 300 samples for total number.

From Table 1, we can see that multiple random forest design is one of key discussed contents of this study. In Step 4, RFs model is established to evaluate the significance of vibration feature. Then, the optimal RFs model is designed for distinguishing the type of HVCB faults.

### 2.2. Vibration Signal Acquisition System with HVCB

**Figure 2:**
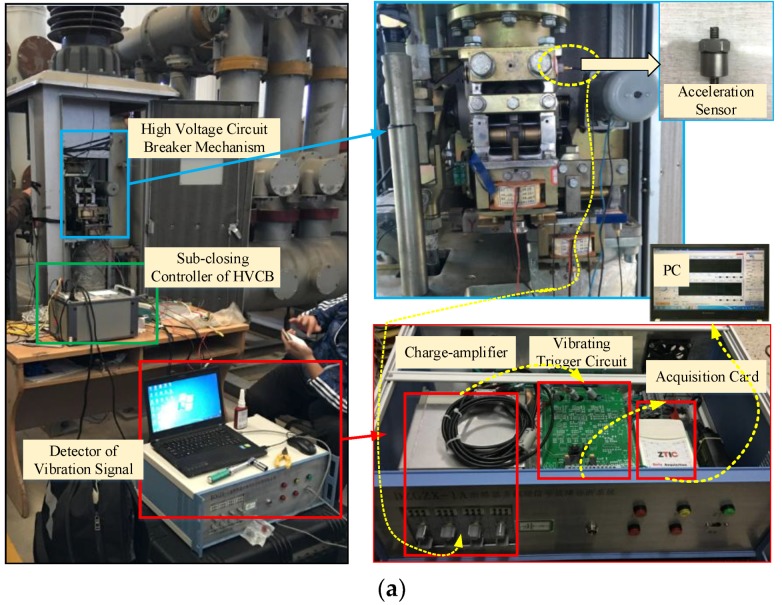

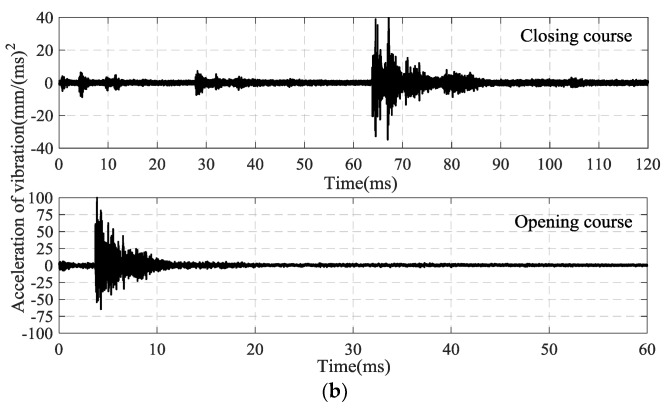
(**a**) Acquisition system with HVCB; (**b**) The measured vibration signal at normal condition.

LW30-252 typed SF_6_ HVCB (Shandong Taikai High Voltage Switchgear Co, Ltd, Shandong, China) is the object of this study. The HVCB is equipped with CT26 spring-operated mechanism of which the divide-shut brake is controlled by alternating electromagnet, the clearance between open contacts is 159 mm, and over-travel is 31 mm. As shown in Figure 2a, the vibration signal acquisition system consists of an acceleration sensor YD-111T, a charge-amplifier TS5863, vibrating trigger circuit and data acquisition card EM9118B. The yellow dotted line represents the direction of vibration signal data flow, which is finally stored in a PC. The YD-111T piezoelectric acceleration sensor has measuring range of ±10,000 g (g = 9.8 m/s^2^), sensitivity of 0.5 mV/g, natural frequency of 45 kHz, frequency response of 15 kHz, a maximum output voltage of ±5 V, and a weight of 10 g.

Previous research has reported that the accuracy of vibration feature-based fault diagnosis method is affected by installation site and installation way of acceleration sensor. In this paper, the acceleration sensor was installed by threaded fastening method. Through measuring the vibration signals at multiple sites, the installation site at Figure 2a is finally selected as the optimal installation site.

When the circuit breaker receives a closing (opening) command, the acquisition system starts sampling. The sampling rate is 300 kHz and the sampling period is 120 (60) ms. In the closing (open) course, a normal vibration signal is shown in Figure 2b.

As shown in Figure 2, the closing process of HVCB involves multi-stage impact and attenuation process, with the maximum vibration acceleration of about 4000 g. In contrast, the opening process is only represented by trumpet-type envelope, with maximum vibration acceleration almost reaching 10,000 g. According to the operation mechanism of HVCB, it can be known that some components of HVCB, such as closing spring and oil damper, impose influence only during closing process. According to the representation form of vibration signal during opening and closing process, the signal during closing process is relatively more abundant. Therefore, the vibration signal during closing process is adopted for HVCB fault diagnosis in this study.

## 3. Feature Extraction

### 3.1. Time and Frequency Analysis Based WPT

From the perspective of time domain, people can easily realize the description of a signal, but apparently this description is not complete. Jean Baptiste Joseph Fourier (1768–1830), a French mathematician, developed a method of expressing any function in terms of a weighted sum of cosines and sines functions [13]. Therefore, we analyzed real-world signals in both time and frequency domain. Based on Fourier transform, any finite energy signal can be represented as Equation (1).

(1) $$\mathrm{FT}\{x(t)\}=X(\Omega )=\int_{-\infty }^{\infty }x(t)e^{-j\Omega t}dt,\Omega =2\pi f$$

where *f* is the frequency in Hertz, and *Ωt* is the phase in radian.

We distinctly discover that Fourier transform theory could not effectively analyze the signal’s spectral variations during this interval of time, which was further confirmed by the vibration signal of HVCB in Figure 1. To avoid this lack of locality property, wavelet transform can be used as an effective method to preserve signal characteristics. However, WT does not split the high frequency bands where the modulation information of vibration signal always exists. A better representation of signal is to extend WT to WPT.

WPT can be implemented by a pair of low-pass and high-pass wavelet filters, denoted as *h*(*k*) and *g*(*k*) = (−1)*^k^h*(1 − *k*), respectively. These filters, also known as Quadrature Mirror Filters (QMF) [14,15], are constructed from the selected wavelet function *ψ*(*t*). The corresponding scaling function *ϕ*(*t*) is described as follow:

(2) $$\{\begin{array}{c} u_{2n}(t)=\sqrt{2}\sum_{k}h(k)u_{n}(2t-k)\\ u_{2n+1}(t)=\sqrt{2}\sum_{k}g(k)u_{n}(2t-k) \end{array}$$

where $u_{0}(t)=\phi (t)$ and $u_{1}(t)=\psi (t)$. Correspondingly, the signal is decomposed as

(3) $$\{\begin{array}{c} d_{j+1,2n}=\sum_{m}h(m-2k)d_{j,n}\\ d_{j+1,2n+1}=\sum_{m}g(m-2k)d_{j,n} \end{array}$$

where *d_j_*_,*n*_ denotes the wavelet coefficients at the *j*-th level and the *n*-th sub-band; *d_j_*_ + 1, 2*n*_ and *d_j_*_ + 1, 2*n* + 1_ denote the wavelet coefficients at the (*j* + 1)-th level and 2*n*-th sub-band, and the wavelet coefficients at the (*j* + 1)-th level and the (2*n* + 1)-th sub-bands, respectively; *m* is the number of the wavelet coefficients.

**Figure 3:**
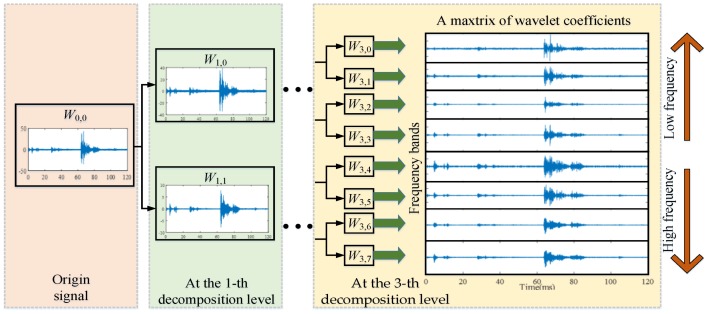
Schematic diagram of wavelet packet time-frequency analysis of vibration signal.

As illustrated in Figure 3, an origin vibration signal can be decomposed by a 3-level binary tree wavelet packet into eight sub-bands. Therefore, the reconstructed signal of wavelet coefficients $W_{j}^{n}$ of each sub-band covers one eighth of the frequency information.

According to Equations (2) and (3) and the wavelet packet time-frequency analysis principle of vibration signal in Figure 3, the time-frequency processing of vibration signal of HVCB was carried out. Using db3 wavelet packet, each vibration signal was subjected to 7-level binary wavelet packet decomposition and wavelet coefficient reconstruction. The system sampling frequency was 300 kHz. The width of each of generated 2^7^ frequency bands was 1.17 kHz. Considering the vibrating sensor YD-111T had a frequency response range from 0 to 15 kHz, the No. 0~12 wavelet coefficients at the 7th level wavelet packet were employed to characterize the time-frequency characteristics of vibration signal.

### 3.2. Feature Extraction

**Figure 4:**
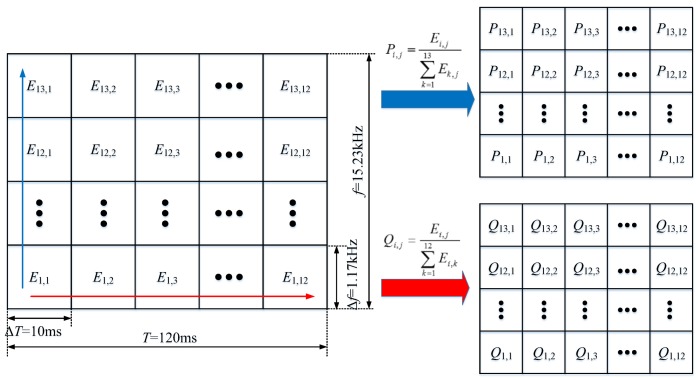
Calculation of wavelet packet time-frequency feature of vibration signal.

According to the wavelet packet time-frequency analysis results in Section 3.1 and regarding Δ*f* = 1.17 kHz, Δ*T* = 10 ms as criteria, the vibration signal during closing process was split into 13 × 12 signal segments, and the energy of each signal segment was calculated using $E=\sqrt{\sum_{t=t_{0}}^{t_{N}}x^{2}(t)}$, as shown in Figure 4. In addition, considering the dispersity of vibration signal energy of HVCB and corresponding normalization processing, the proportion of each signal segment in time-frequency direction was calculated using Equation (4). *P_i_*_,*j*_ and *Q_i_*_,*j*_ represent the proportion of the *j*-th signal in frequency direction within the *i*-th period of time as well as the proportion of the *i*-th signal in time direction within the *j*-th frequency band, respectively.

(4) $$\{\begin{array}{l} P_{i,j}=E_{i,j}/\sum_{k=1}^{13}E_{k,j}\\ Q_{i,j}=E_{i,j}/\sum_{k=1}^{12}E_{i,k} \end{array}$$

In the information theory, Shannon entropy is the most common measure for evaluating the confusion degree of a signal. In fact, it is usually described as an essential feature. Shannon entropy Θ is defined as:

(5) $$\Theta =-\sum_{i=1}^{N}\varsigma_{i}\log\varsigma_{i}$$

where *ζ_i_* is the probability of random event *Υ* = *y_i_* and $\sum_{i=1}^{N}\varsigma_{i}=1$. When *ζ_i_* = 0, there is a convention that $\varsigma_{i}\log\varsigma_{i}=0$ [20]. According to Equations (4) and (5), the Shannon entropy feature space $\mathrm{WTFE}=[\mathrm{WTFE}f_{t},\mathrm{WTFE}t_{f}]$ of wavelet packet analysis matrix can be obtained, as shown in Equation (6).

(6) $$\{\begin{array}{c} \mathrm{WTFE}f_{t}=[-\sum_{j=1}^{12}(P_{1,j}\times \ln(P_{1,j})),-\sum_{j=1}^{12}(P_{2,j}\times \ln(P_{2,j})),\ldots ,-\sum_{j=1}^{12}(P_{13,j}\times \ln(P_{13,j}))]\\ \mathrm{WTFE}t_{f}=[-\sum_{i=1}^{13}(Q_{i,1}\times \ln(Q_{i,1})),-\sum_{i=1}^{13}(Q_{i,2}\times \ln(Q_{i,2})),\ldots ,-\sum_{i=1}^{13}(Q_{i,12}\times \ln(Q_{i,12}))] \end{array}$$

**Figure 5:**
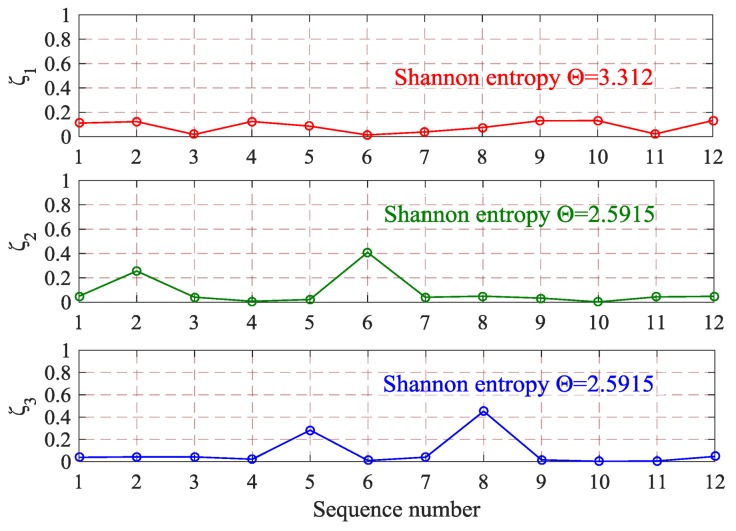
Incomplete description of Shannon entropy.

Shannon entropy is a type of characteristic description of signal. However, this description of signal is not complete. Taking the three sequences {*ζ*_1_, *ζ*_2_, *ζ*_3_} in Figure 5 as example, form the analysis perspective of Shannon entropy, only the difference between red sequence and other two sequences can be distinguished, i.e., the entropy value is different. Although the green curve and blue curve share identical entropy value (indicating identical dispersity of the sequence), the difference in sequence order still cannot be characterized. Therefore, in the process of feature extracting, the difference in distribution order of sequence was considered and wavelet packet analysis-based time-frequency energy was regarded as HVCB vibration signal feature to form feature space WTFER, as shown in Equation (7).

(7) $$\mathrm{WTFER}=[{\mathrm{WTFER}}_{t},{\mathrm{WTFER}}_{f}]=[P_{1,1},P_{1,2},\ldots P_{1,12},P_{2,1},\ldots ,P_{13,12},Q_{1,1},\ldots ,Q_{13,12}]$$

**Figure 6:**
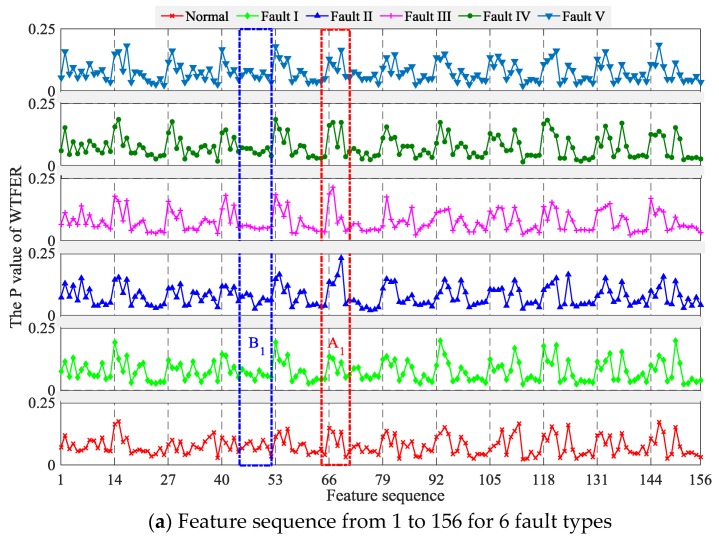

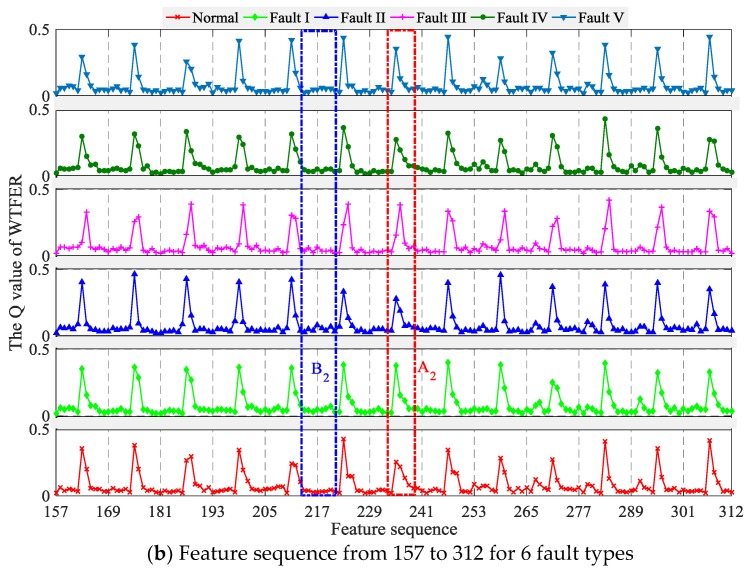
Feature description. A1, A2, B1, B2 represents a length of feature sequence (65~71th, 233~239th, 44~52th, 214~222th feature sequence, respectively).

According to Equations (4) and (7), the feature sequence of HVCB vibration signal under different faults was calculated, as shown in Figure 6. It can be seen from the figure that the respective dispersities of feature sequences are identical, and Shannon entropy values are similar to each other. By comparing the feature sequences of different fault samples, it can be seen that there is a large amount of feature variables with no difference or insignificant differences, as shown in A_1_ and A_2_ of Figure 6. The classifier can make a good classification according to the differences among these features. Meanwhile, there is a large number of uncorrelated features in feature vector, as shown in B_1_ and B_2_ of Figure 6, which may affect subsequent classifier performance. Therefore, it is very important to reduce feature space dimension or to select a diagnosis algorithm with high accuracy and robustness.

## 4. Fault Diagnosis Based on Random Forest

### 4.1. Classification and Regression Tree

**Figure 7:**
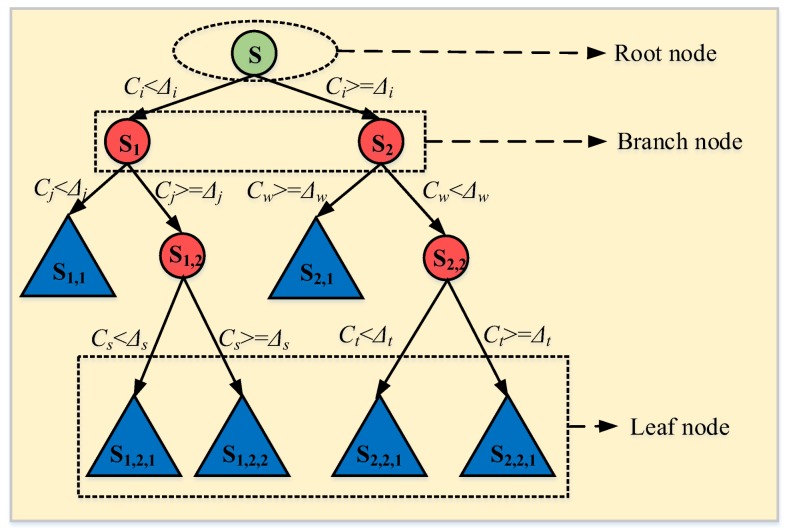
Model representation as a binary tree.

For classification or regression matters, a decision tree is a highly interpretable method with graph description [28] based on partition of the feature space. In general, the growth of a decision tree typically starts at the root node. When the data sample set is not pure, a series of binary tree splitting will happen until the data sample set is pure. The purity of sample data set refers to the proportion of similar samples within a node phase. Based on the value of random one of the explanatory feature variables, two child nodes are generated. The splitting process is persisted endlessly. Ultimately, a terminal node or leaf is reached, and a decision tree model is given, as illustrated in Figure 7.

It is the most important core for the whole algorithm to define the impurity of each node and minimize this impurity with reasonable partitioning. In principle, *Gini* index is a better criterion by which node impurity is minimized in a classification problem [38]. Suppose that there is a random variable *x* with a total of *K* states, each indexed by *k*; and *p_k_* is the probability of *x* belonging to state *k*. Then, the *Gini* index can be expressed as

(8) $$Gini(x)=\sum_{k=1}^{K}p_{k}(1-p_{k})=1-\sum_{k=1}^{K}p_{k}^{2}$$

In a node of classification and regression tree (CART), if a partition point *Δ_i_* and the *i*-th attribute *C_i_* in feature space are selected, the training data set **S** is divided into two parts, namely **S_1_** and **S_2_**, then the *Gini* index of CART at a node can be calculated as

(9) $$Gini(S,C_{i})=\frac{\left|S_{1}\right|}{\left|S\right|}Gini(S_{1})+\frac{\left|S_{2}\right|}{\left|S\right|}Gini(S_{2})$$

*Gini*(**S**,*C_i_*) represents the non-determinacy or impurity of training data set **S** by the partition point of *C_i_* = *Δ_i_*. As the *Gini* index increases, the purity of data set reduces, and the result of splitting becomes increasingly worse. Therefore, this iterative procedure of binary partition searches the least *Gini* index throughout all possible values within the definition domain of all attribute variables, and finally a whole CART is formed, as shown in Figure 7.

### 4.2. Random Forest

The vibration information description of HVCB under the same fault is of large dispersity, i.e., the noise ratio is large. The decision tree-based techniques are limited by high variance and instability, so the classification accuracy may be greatly impacted by the noise. When uncorrelated feature variables are too high, the performance of an individual decision tree becomes worse. It is well known that individual classifiers’ performance can be improved by an ensemble method that integrates a set of classifiers [32]. The most popular ensemble technique is bagging tree (BT), which proceeds by generating bootstrap samples from the original data set, constructing a CART from each bootstrap sample, and voting to combine. The majority voting rule is given by [40]

(10) $$L=\arg_{L}^{\max}\sum_{j=1}^{N_{tree}}I(H_{j}(C_{i})==L_{k})$$

where *I*(**C***_i_*) is an indication function; arg() is a value function representing the number of trees that classify the test sample as the classification vector *L*.

**Figure 8:**
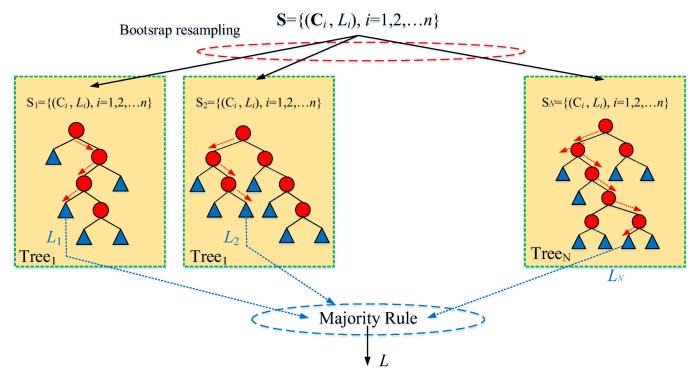
Random forest structure.

The main principle behind bagging’s performance is that the averaging reduces the variance of individual classifiers without increasing their bias. Bagging generally performs well with unstable base learners for whom small changes in the training data can lead to large changes in the learned model [32]. As an improved bagging tree method, random forest (RF) [35] randomly selects large amounts of attributes at each node of CART. Hence, RF has faster training speed, higher prediction accuracy and stronger robustness than CART and BT. Beyond that, based on bagging concept, RF provides an out of bagging (OOB) data-based unbiased estimate of test set error. OOB data are also used to estimate the importance of variables. These two estimates (test set error estimate and variable importance) are the most useful byproducts of RF. The structure of RF model is shown in Figure 8.

To increase classification accuracy, low bias of CART and low correlation of CARTs in forest are essential. To achieve low bias, each CART in forest grows to maximum depth. To obtain low correlation, randomization is applied in two aspects [37]: (1) CART is grown on a bootstrap sample of the training feature set; (2) *m* characteristic attributes are randomly selected out of *d* available. In general, the parameter *m* is set to $\sqrt{d}$ for classification problem. Table 2 describes the procedure of the RF algorithm.

**Figure 9:**
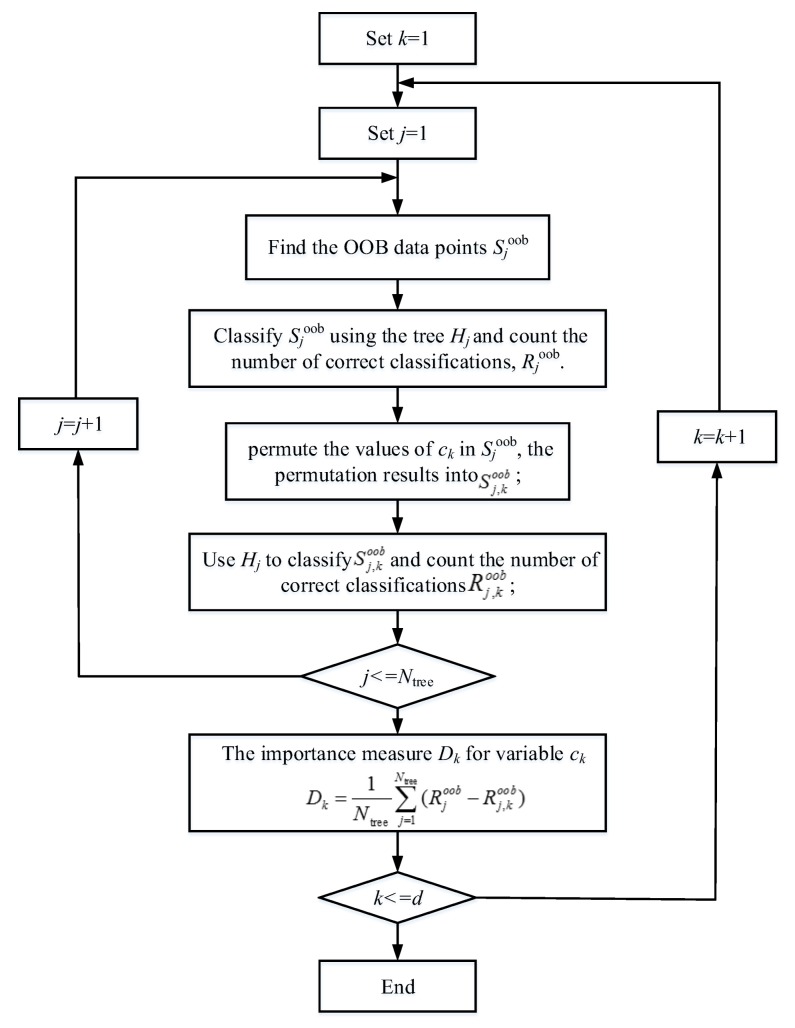
The process of analyzing the importance of feature based on random forest algorithm.

As indicated in various literature, a feature space dimension higher is by no means better during classification process. If there is too much non-essential features during feature extracting process, the main feature may be overwhelmed, which may lead to diagnosis bias. Therefore, much research on accurate estimation of variable importance has been conducted [37]. With the aid of OOB and random forest algorithm, the importance of variables in feature space was estimated. The detailed process of analyzing the importance of the *k*-th feature in feature space is shown in Figure 9.

As shown in Figure 9, the analysis of feature importance can effectively reduce the dimension of feature space and decrease the impact of non-essential features on diagnosis. In addition, by reducing the dimensions of feature space, the training efficiency and diagnosis accuracy of the diagnostic model can be significantly improved. This paper proposes a strategy for optimizing feature space strategy based on the above estimation process, of which the pseudo-code is shown in Table 3. The RF_1_ was established based on original feature space and the error rate of OOB sample was recorded. According to the analysis of feature importance rank in Figure 9, top *d* × (1 − *λ*) important features were adopted to constitute a new feature space, and the second-generation RF model was constructed based on this new feature space to record the error rate. After that, through analyzing the feature importance rank in such feature space, top *d* × (1 − *λ*) important features were selected again to constitute another new feature space. This process was repeatedly performed until the feature space dimension was less than *δ*. The feature space with minimum OOB error rate was adopted for the description and the RF model was employed as the optimal diagnosis model.

## 5. Experimental Result and Analysis

Based on the description of HVCB vibration data samples (including training and test sample) and fault type in Section 2, feature extracting process based on vibration signal time-frequency analysis in Section 3, and the design flow of robust random forest algorithm and feature space optimization based on ensemble learning in Section 4, this section verifies the superiority of the proposed method by comparing the diagnostic performance and optimization results of different classification models with original feature space and optimized feature space.

### 5.1. Diagnosis Results with the Origin Feature Space

**Figure 10:**
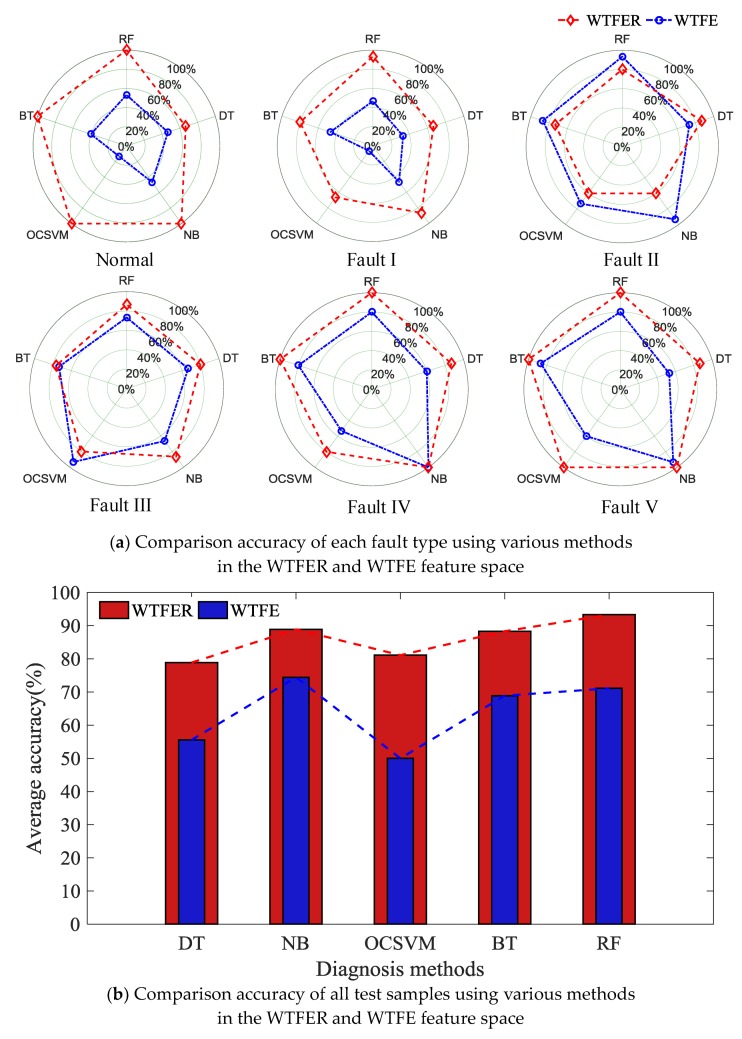
Diagnosis results based on WTFER feature space.

For each fault condition, there were 50 vibration signal samples. The 50 samples for each fault condition were randomly divided into two parts: training sample set consisting of 35 samples and test sample set consisting of 15 samples. After that, based on training sample set, WTFE and WTFER were selected as the input feature vectors of diagnostic model to train the parameters of different classifier models (DT, NB, OCSVM, BT and RF) Finally, based on test sample set, we compared the diagnosis accuracy rate $TP_{i}/\left(TP_{i}+FP_{i}\right)$ for single fault with the averaged diagnosis accuracy rate $\sum_{i}^{n_{c}}TP_{i}/\sum_{i}^{n_{c}}(TP_{i}+FP)$ for all faults, as shown in Figure 10, Table 4 and Table 5. Where *TP_i_* (*FP_i_*) denotes the number of true (false) positive instances in the *i*-th fault and *n_c_* is the number of the fault categories.

According to Figure 10 and Table 4 and Table 5, it can be known that (1) despite any classifier model, the diagnosis accuracy with WTFER parameter as feature vector input is always significantly higher than that with WTFE parameter as input. This indicates the Shannon entropy of signal is an incomplete description of original signal feature, which will greatly impact on diagnosis accuracy. Selecting a proper feature space is beneficial for improving diagnosis accuracy of model; (2) In spite of analysis from diagnosis accuracy of single fault or from average diagnosis accuracy of all faults, the classifier model based on integrated method (BT and RF) always has higher diagnosis accuracy (93.33%) than that of single classifier model (DT-78.89%, NB-88.89% and OCSVM-81.11%), indicating the superiority of integrated learning method.

### 5.2. Diagnosis Results with the Optimal Feature Space

**Figure 11:**
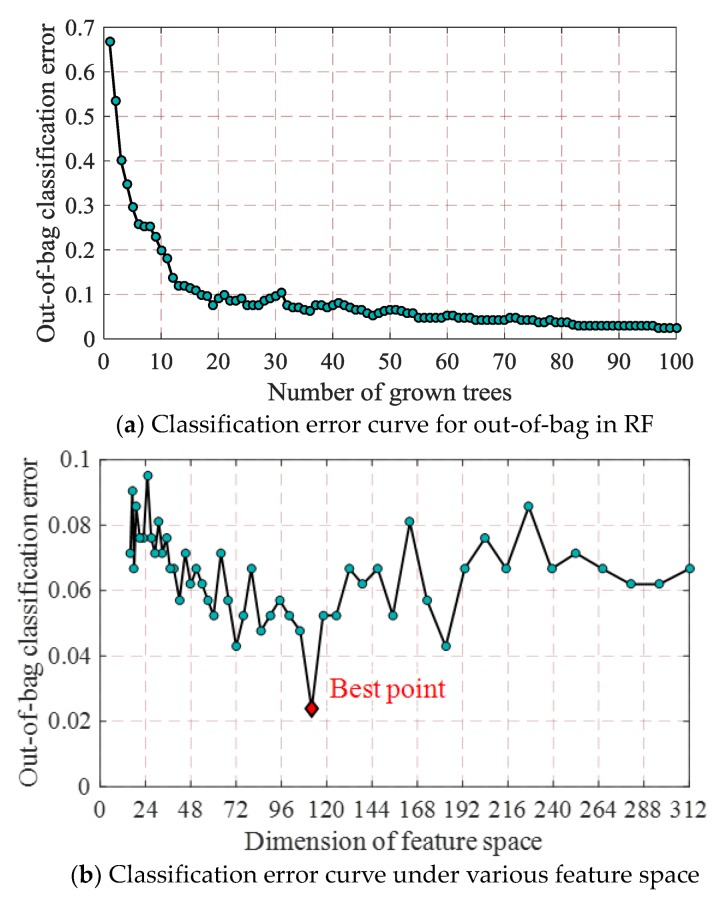

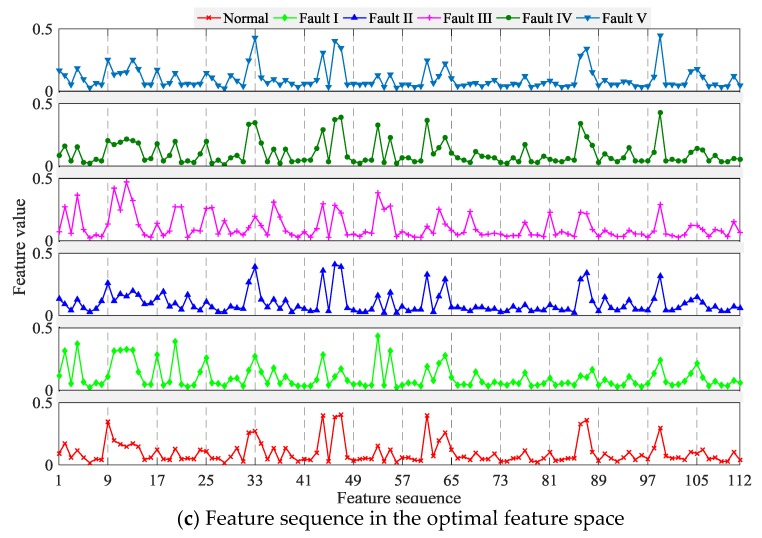
Calculation results of parameters of optimized feature space.

Using the random forest model based on WTFER feature space in Section 5.1 and the optimization flow of feature space in Table 3, we optimized the input feature vector of classifier model, realized the description of optimized feature space, and designed optimized random forest classifier model of which the OOB error curve is shown in Figure 11a. The error curves of OOB data diagnosis of random forest under different feature spaces are shown in Figure 11b. Under optimized feature space, the comparisons of feature vectors of different fault samples are shown in Figure 11c.

As shown in Figure 11a, the OOB error of random forest under optimized feature space gradually decreases and tends to be stable, indicating that the model has sound diagnosis performance. As shown in Figure 11b, as the feature space dimension decreases, the OOB error of random forest does not display monotonical increasing or decreasing trend, but there exists an optimized feature space (a point where minimum OOB error locates), i.e., the feature space constituted by top 112 important features. By comparing Figure 11c with Figure 6, it can be known that the optimization process greatly reduced low-correlated variables, decreased the dimension of feature space, so that the reserved features can indicate the difference among faults more clearly.

**Figure 12:**
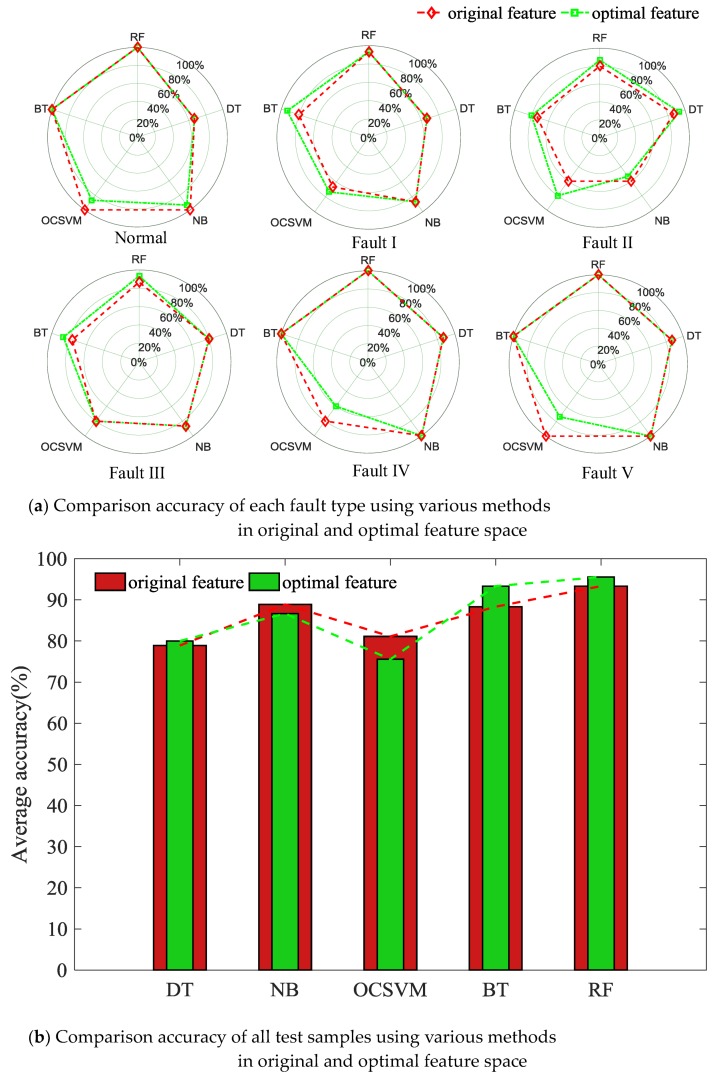
Diagnosis results after feature space optimization.

Each classifier model was reconstructed based on optimized feature space. The diagnosis results of different models after feature space optimization were compared based on vibration signal test set, as shown in Figure 12 and Table 6.

By comparing Figure 10 and Figure 12, it can be known that (1) after feature space optimization, the diagnosis accuracy of the random forest model increases to 95.56%, which is significantly higher than that of other classifier models; (2) under optimized feature space, the diagnosis accuracies of all classifiers are increased, indicating that the proposed optimization process is effective, and the removal of low-correlated feature variables is conductive to the improvement of fault diagnosis results.

## 6. Conclusions

In this work, a novel diagnosis method for HVCB mechanical faults is proposed based on WTFER feature space and random forest model. The experimental results show the superiority of the proposed method in HVCB fault diagnosis. Conclusions can be drawn as follows:

(1) To timely discover potential risk of HVCB, a precise diagnosis method and detection system for the mechanical fault of HVCB is designed based on the vibration signal under different conditions;
(2) As the frequency of HVCB vibration signal changes with time, a time-frequency analysis technique is applied in the feature extraction process. Compared with incomplete descriptions of WTFE based on Shannon entropy, a WTFER feature vector describes the vibration signal characteristics more comprehensively and effectively;
(3) In comparison to the conventional single classification, random forest with bagging technology and random split selection skill achieves better performance and higher accuracy in mechanical fault diagnosis of HVCB;
(4) As one of the most useful byproducts of random forest algorithm, the assessment of feature importance can be used to optimize the feature space for further improving diagnosis precision of the RF model.

Currently, the vibration information-based diagnostic technique for HVCB is still in the phase of artificial feature extraction. With the development of artificial intelligence technology, automatic identification diagnosis of characteristic space description using deep learning will become the research focus in the future. In addition, the fault normally does not occur alone but occurs multiple times during the operation of HVCB; therefore, applying the single-fault diagnosis model in identification of combined faults will become a key research direction for intelligent fault diagnosis of HVCB.

## Figures and Tables

**Table 1:** General procedure of HVCB fault detection by the proposed method.

Step	Description
Step 1	HVCB vibration signals acquisition
Step 2	Seven-layer WPT is used to decompose vibration signals into 128 components with different frequency bands
Step 3	Based on time-frequency description of WPT, the feature space is established using time-frequency energy ratio
Step 4	The RFs diagnosis model is established using feature space
Step 5	Based on RFs model, the importance of vibration signal is sorted, and the feature space is optimized;
Step 6	A new RF diagnosis model is constructed for optimizing feature space;
Step 7	The performance of the optimized RF diagnosis model is validated based on testing samples

**Table 2:** Random forest algorithm.

**Algorithm 1.** Random Forest
**Input:**
Training set of variable fault’s vibration signal, S = {(*C_i_*, *L_i_*), *I* = 1, 2, … *n*}, (C*_i_*, *L_i_*)∈*R^d^* × *R*, (C*_i_*, *L_i_*) represent feature set and label of the *i*-th sampling; Test sample X*_i_*∈*R^d^*;
**Output:**
Collection of trees *T* = {*H*_j_(C*_i_*), *j* = 1, 2, …, *N*_tree_};
**Process:**
For *j* = 1, 2, …, *N*_tree_
(1) The training set S is generated from the original training set with the bootstrap resampling method;
(2) The decision tree is generated based on CART algorithm. *m* characteristic attribute values are randomly selected from *d* characteristic attribute values at each node of decision tree;
(3) The best characteristic value is selected from *m* characteristic attribute values at each node according to the *Gini* index;
(4) A non-pruned tree *H_j_* is generated;
(5) Splitting until the tree grows to the maximum, and then random forest is generated
(6) End.
Test sample X*_i_* is introduced into random forest and diagnosis result is recorded.

**Table 3:** Pseudo-code for optimizing feature space.

**Input:** S = {(C*_i_*, *L_i_*), *i* = 1, 2, … *n*}, (C*_i_*, *L_i_*)∈*R^d^* × *R*, (C*_i_*, *L_i_*)RF parameters, minimum feature space dimension *δ*, reduce rate *λ*.
1. **Function**
2. *m* = 1, design RF*_m_* with S, calculate and sort *D_k_*,*_m_* based on RF*_m_*;
3. **While** *d* > *δ*
4. *m* = *m* + 1
5. S*_m_* ←select the top *d* × (1 − *λ*) important features to form new feature space S*_m_* and *d* = *d* × (1 − *λ*)
6. RF*_m_* ←RF*_m_ based on* S*_m_*;
7. {*D_k_*,*_m_*, E_oob,*m*_} ←calculate *D_k_*,*_m_* and error rate of OOB(E_oob,*m*_);
8. **End**
9. **Return** min({E_oob,*m*_})→RF*_m_*
10. **End function**

**Table 4:** WTFE-based diagnosis results.

Fault Type	Accuracy Rate				
	DT	NB	OCSVM	BT	RF
Normal	46.67%	46.67%	13.33%	40%	53.33%
Fault I	33.33%	46.67%	6.67%	46.67%	46.67%
Fault II	73.33%	93.33%	73.33%	86.67%	93.33%
Fault III	66.67%	66.67%	93.33%	73.33%	73.33%
Fault IV	60%	100%	53.33%	80%	80%
Fault V	53.33%	93.33%	60%	86.67%	80%
Total	55.56%	74.45%	50%	68.89%	71.11%

**Table 5:** WTFER-based diagnosis results.

Fault Type	Accuracy Rate				
	DT	NB	OCSVM	BT	RF
Normal	66.67%	100%	100%	100%	100%
Fault I	66.67%	86.67%	66.67%	80%	93.33%
Fault II	86.67%	60%	60%	73.33%	80%
Fault III	80%	86.67%	80%	86.67%	86.67%
Fault IV	86.67%	100%	80%	100%	100%
Fault V	86.67%	100%	100%	100%	100%
Total	78.89%	88.89%	81.11%	90%	93.33%

**Table 6:** Diagnosis results after feature space optimization.

Fault Type	Accuracy Rate				
	DT	NB	OCSVM	BT	RF
Normal	66.67%	93.33%	86.67%	100%	100%
Fault I	66.67%	86.67%	73.33%	93.33%	93.33%
Fault II	93.33%	53.33%	80%	80%	86.67%
Fault III	80%	86.67%	80%	86.67%	93.33%
Fault IV	86.67%	100%	60%	100%	100%
Fault V	86.67%	100%	73.33%	100%	100%
Total	80%	86.67%	75.56%	93.33%	95.56%

## Nomenclature

HVCB	High-voltage circuit breakers
DT	Decision tree algorithm
NB	Naive bayes algorithm
OCSVM	One class support vector Machine
BT	Bagger decision tree algorithm
RF	Random forest
OOB	Out of bagging
WPT	wavelet package transform
WTFE	wavelet time-frequency entropy
WTFER	wavelet time-frequency energy rate
*Ei*,*j*	energy in the *i*-th time and *j*-th frequency section
*Pi*,*j*	energy rate alone frequency direction
*Qi*,*j*	energy rate alone time direction
**S**	training sample set
**X**	test sample set
**C** *_i_*	*i*-th sample feature ∈ **R***^d^*,
*d*	feature space dimension
*k*	*k*-th feature in feature space
*L_i_*	*i*-th sample label (Fault type) ∈ R
*H_j_*	*j*-th decision tree
*T*	collection of trees represent forest
*N*_tree_	number of tree in the forest
*S_j_* ^oob^	OOB data set in *j*-th decision tree
*S_j,k_* ^oob^	OOB data set that permuting *k*-th feature in *S_j_*^oob^ for *j*-th decision tree
*R_j_* ^oob^	number of correct classification in *j*-th decision tree with OOB data
*R_j,k_* ^oob^	number of correct classification in *j*-th decision tree for *S_j,k_*^oob^
*D_k_*	important measure for *k*-th feature
*δ*	minimum feature space dimension
*λ*	reduce rate
RF*_m_*	*m*-th random forest model
*E*_oob,*m*_	error rate of OOB data in *m*-th random forest model
